# High IFN-γ Release and Impaired Capacity of Multi-Cytokine Secretion in IGRA Supernatants Are Associated with Active Tuberculosis

**DOI:** 10.1371/journal.pone.0162137

**Published:** 2016-09-07

**Authors:** Séverine Carrère-Kremer, Pierre-Alain Rubbo, Amandine Pisoni, Sophie Bendriss, Grégory Marin, Marianne Peries, Karine Bolloré, Dominique Terru, Sylvain Godreuil, Arnaud Bourdin, Philippe Van de Perre, Edouard Tuaillon

**Affiliations:** 1 UMR1058 INSERM/University Montpellier/EFS, Montpellier, France; 2 University Montpellier, Montpellier, France; 3 CHRU Montpellier, Departments of Bacteriology-Virology, Montpellier, France; 4 CHRU Montpellier, Department of Pneumology, Montpellier, France; 5 CHRU Montpellier, Department of Medical Information, Montpellier, France; Hopital Raymond Poincare - Universite Versailles St. Quentin, FRANCE

## Abstract

Interferon gamma (IFN-γ) release assays (IGRAs) detect *Mycobacterium tuberculosis* (*Mtb)* infection regardless of the active (ATB) or latent (LTBI) forms of tuberculosis (TB). In this study, *Mtb*-specific T cell response against region of deletion 1 (RD1) antigens were explored by a microbead multiplex assay performed in T-SPOT TB assay (T-SPOT) supernatants from 35 patients with ATB and 115 patients with LTBI. T-SPOT is positive when over 7 IFN-γ secreting cells (SC)/250 000 peripheral blood mononuclear cells (PBMC) are enumerated. However, over 100 IFN-γ SC /250 000 PBMC were more frequently observed in the ATB group compared to the LTBI group. By contrast, lower cytokine concentrations and lower cytokine productions relative to IFN-γ secretion were observed for IL 4, IL-12, TNF-α, GM-CSF, Eotaxin and IFN-α when compared to LTBI. Thus, high IFN-γ release and low cytokine secretions in relation with IFN-γ production appeared as signatures of ATB, corroborating that multicytokine *Mtb*-specific response against RD1 antigens reflects host capacity to contain TB reactivation. In this way, testing cytokine profile in IGRA supernatants would be helpful to improve ATB screening strategy including immunologic tests.

## Introduction

Almost one third of the world population is infected by *Mycobacterium tuberculosis* (*Mtb*). Most of the infected individuals will remain asymptomatic, but 10% will develop active tuberculosis (ATB) disease during lifetime leading to death if left untreated [1]. There is no diagnostic gold standard for latent tuberculosis infection (LTBI). It is characterized by a specific immune response directed against *Mtb*, using tuberculin skin test (TST) or interferon-gamma (IFN-γ) release assays (IGRAs), when there is no clinical, bacteriological and radiological evidence of ATB. IGRAs are dedicated to the diagnosis of *Mtb* infection based on the assessment of IFN-γ secretion by *Mtb-*specific T cells in response to RD1 antigen stimulation [2]. Two commercial IGRAs are available: i) the T-SPOT.TB assay (T-SPOT, Oxford Immunotech) is an enzyme-linked immune-spot assay that enumerates IFN-γ-secreting cells (SC) after stimulation of PBMC (Peripheral Blood Mononuclear Cells) with *Mtb* peptides derived from ESAT-6 (Rv3875) and CFP-10 (Rv3874) antigens; ii) the QuantiFERON Gold In-Tube assay (QFT, Qiagen) quantifies IFN-γ released after incubation of whole blood with a cocktail of peptides derived from ESAT-6, CFP-10 and TB7.7 (Rv2654c) antigens. Both tests are qualitative: T-SPOT is positive over 7 IFN-γ SCs/250 000 PBMC and QFT over 0.35 IU/ml. IGRAs have a good specificity, close to 100%. They detect T cell immunity induced by *Mtb* infection but not by most atypical mycobacteria or by Bacillus-Calmette-Guérin vaccine [2]. IGRAs’ sensitivity is estimated to 80–90%, however it is diminished by HIV infection and in children [2].

To better characterize the immune response in ATB versus LTBI individuals, analysis of multicytokine production following *Mtb* antigen stimulation can be performed in addition to IFN-γ detection. Previous reports indicated that interleukin-2 (IL-2), 10 kDa IFN-γ-inducible protein (IP-10 or CXCL-10) and monokine induced by IFN-γ (MIG or CXCL9) may be surrogate markers for detecting subjects with a non-replicating *Mtb* infection [3–10]. We also reported that evaluation of IL-2, IL-15, IP-10 and MIG) may be useful to detect LTBI in healthcare workers exposed to TB and tested positive by TST but negative by IGRAs [11].

Furthermore, polyfunctional T cell profiles play a key role in controlling chronic virus infections and intracellular pathogens [12,13]. High frequencies of polyfunctional *Mtb* specific T cells producing combination of IFN-γ, tumor necrosis factor-α (TNF-α) and IL-2 may be the hallmark of LTBI [12–15]. By contrast, low secretion of IL-2 compared to IFN-γ production or high frequency of single-positive TNF-α *Mtb*-specific T cells have been associated with defect in the control of TB infection [12–15]. These results suggest that a shift from IL-2 to IFN-γ by T cells occur during progression from latent stage to disease.

In this study, to characterize T cell responses associated with ATB or LTBI in a low incidence context, we quantified several cytokines secreted along with IFN-γ in IGRA supernatants. Investigations were conducted to prospectively assess the cytokine signatures using the more sensitive IGRA, T-SPOT, combined with a microbead multiplex assay, a powerful tool that simultaneously measures multiple analytes at a time.

## Materials and Methods

### Data collection and participants

This study was conducted in the Montpellier University Hospital (France) on outpatients and hospitalized adults. Demographic and clinical characteristics of the patients are detailed in Table 1. Patients were included after approval of the local ethics committee (Sud-Méditerrannée-III, France, AFSSAPS n° 2010-A00422-37) and after providing a written informed consent. Subjects were enrolled on a positive IGRA result and on suspicion of ATB or LTBI based on clinical presentation. The status ATB versus LTBI was established on *Mtb* culture results, and/or computed tomography scan, and/or clinical conclusions.

**Table 1:** Clinical characteristic of 150 subjects included in this study.

Subjects tested by T-SPOT	ATB group	LTBI group
Numbers of patients	35	115
Median age in year (IQR)	39 (27–66)	47 (33–66)
Number of females (%)	11 (30%)	48 (42%)
Median T-SPOT results (IQR)	115 (50–163)	41(20–105)
Smear positive	10	0
Culture positive	27	0

### IGRA

The T-SPOT.TB assay was carried out according to the manufacturer’s instructions (Oxford Immunotec Ltd, Abingdon, UK). The higher result for either ESAT-6 or CFP-10 stimulation was considered for further analysis. Remaining cell-free supernatants were stored at −20°C until cytokines quantification.

### Cytokine profile

Cytokine secretion was quantitated in T-SPOT cell-free culture supernatants using a microbead-based multiplex method (Human cytokine 25-plex panel; Invitrogen, Villebon sur Yvette, France) and a Luminex 100 apparatus (Luminex, Oosterhout, The Netherlands) as previously described [11]. Cytokine levels were obtained by subtraction of the value in unstimulated microwell from the value obtained following ESAT-6 or CFP-10 stimulation. Cytokine indexes were calculated based on cytokine concentration/IFN-γ spot number*100.

### Design of the study

The cytokine signatures were explored using the T-SPOT assay combined with a microbead multiplex assay to quantify cytokine secretion in cell culture supernatants. Levels of secretion were analysed after ESAT-6 stimulation. Levels of secretion following CFP-10 stimulation were used to confirm results obtained with ESAT-6.

### Statistical analysis

Cytokine values located below the detection level of the assay were considered as undetected and excluded from the analysis. Results were analysed using GraphPad Prism 6 (GraphPad Software, La Jolla, CA, USA), SAS 9.4 (SAS/STAT Software, Cary, NC, USA) and R 3.1.1 (R Foundation for Statistical Computing, Vienna, Austria). The median cytokine levels (interquartile ranges (IQR)) of the ATB and LTBI groups were compared using the nonparametric Wilcoxon Mann-Whitney test. A p-value <0.05 was considered significant. The ROC (Receiver Operating characteristics) curves of the selected cytokines were constructed by plotting the true positive rate (ATB samples; sensitivity) against the false positive (LTBI samples; 1-specificity). Areas under the curve (AUC) were calculated along with their 95% confidence intervals (95% CI). Cut-offs for cytokines were determined using the Youden index, which was defined as sensitivity + specificity-1, and a visual appreciation on scatter plots. With these cut-offs, crude and adjusted odd rations (OR) were calculated using logistic regression. Partial least squares discriminant analysis (PLSDA) was used to determine multivariate cytokine profiles that best distinguish between ATB and LTBI [16,17]. In addition, unsupervised hierarchical classification analyses were carried out in order to analyse potential clusters of ATB patients and whether or not it could be associated with certain cytokine profiles. The analysis results were expressed as heatmaps, computed after scaling the variables.

## Results

### Characteristics of the enrolled individuals

Clinical characteristics of the patients are shown in Table 1. A total of 150 patients tested positive with T-SPOT were included in the first part of the study. Among them, 35 (23%) had an ATB and 115 (77%) a LTBI.

### High number of IFN-γ secreting cells in T-SPOT is associated with ATB

**Fig 1:**
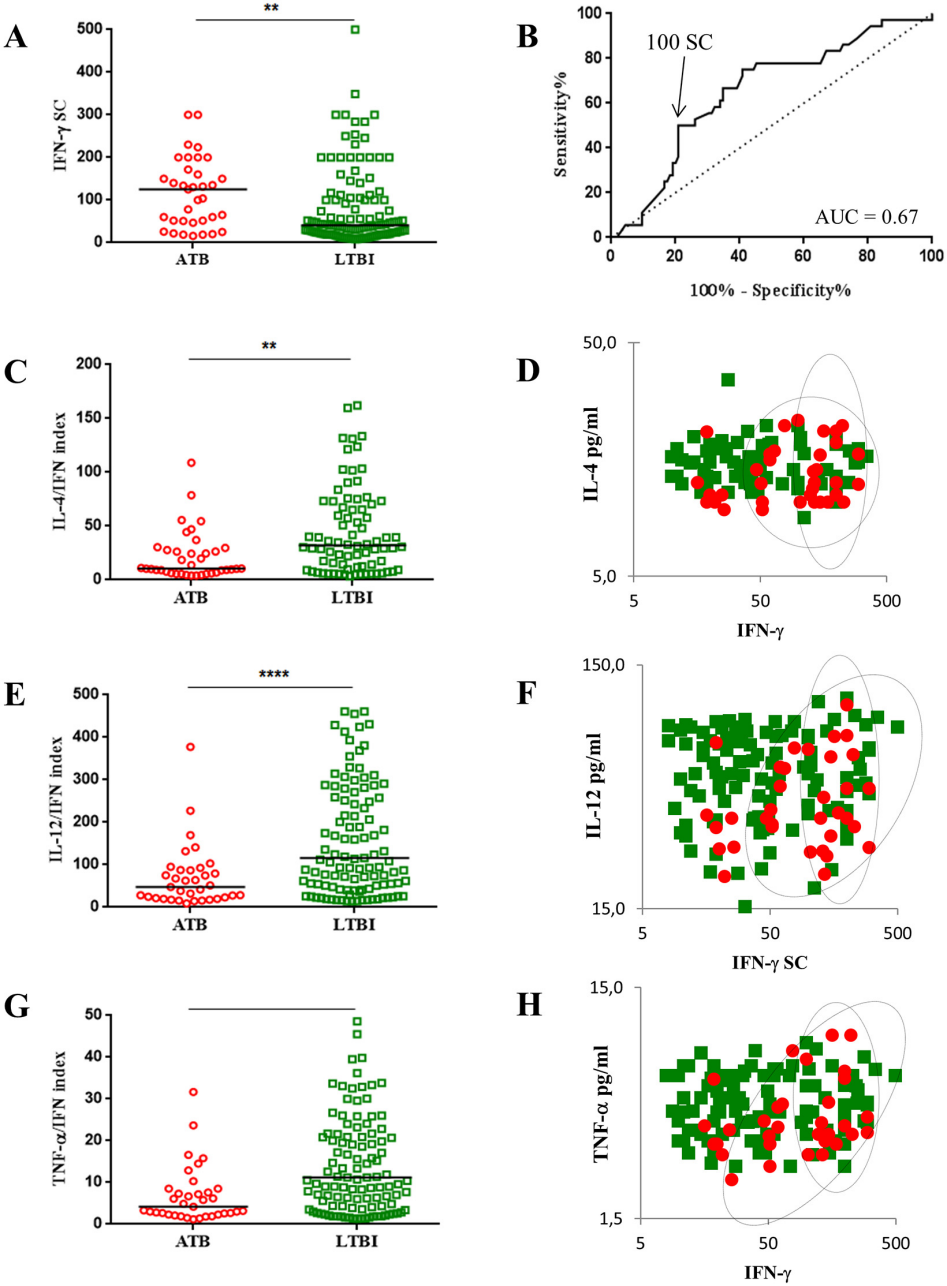
Cytokines response in T-SPOT supernatants. **A-** The expression of IFN-γ in ESAT-6 stimulated T cells was quantified by the T-SPOT assay. Results are expressed by numbers of secreted cells (SC) per 250 000 PBMC for 36 patients with ATB (red circles) and 115 patients with LTBI (green squares). The median is shown in each group. The p value is calculated by the Mann-Whitney U test. ** corresponds to p≤0.01. **B-** The ROC curve displays sensitivity vs specificity for IFN-γ in differentiating the ATB group from the LTBI group. The AUC and a threshold at 100 SC/250000 PBMC are indicated in the graph. **C-E-G-I-K-M-** IL-4, IL-12, TNF-α, GM-CSF, Eotaxin and IFN-α indexes (cytokine/IFN-γ *100), ATB are represented by red circles and LTBI by green squares. The median indexes are shown in each group of each panel. p values are calculated by the Mann-Whitney U test. ** corresponds to p≤0.01, *** to p≤0.001 and **** to p≤0.0001. **D-F-H-J-L-N-** Biparametric graphs between the numbers of IFN-γ immunospots in response to ESAT-6 stimulation and the level of cytokines (pg/ml) in the corresponding supernatants. ATB are represented by red circles and LTBI by green squares. Defined risk groups are circled.

A higher number of IFN-γ SCs was enumerated in patients with ATB compared to patients with LTBI (Fig 1A). The median values were 115 (IQR 48–169) for ATB and 41 (IQR 20–105) for LTBI (p = 0.0055).

Although the T-SPOT is a qualitative assay, its ability to discriminate ATB from LTBI based on the number of IFN-γ SCs was evaluated using a ROC curve (Fig 1B). A poor AUC was obtained, highlighting the low performance of the T-SPOT to identify ATB among patients with *Mtb* infection (0.67 (95% CI: 0.58–0.77)). Based on Youden index, a threshold of 100 IFN-γ-SCs/250.000 PBMC was obtained with a specificity reaching 74% and a sensitivity of 53%. Twenty out of 35 patients (57.1%) from the ATB group and 35 out of 151 patients (23.2%) from the LTBI group had results above this cut-off value. Thus, patients with positive IGRA over 100 IFN-γ-SCs/250 000 PBMC had a three-fold higher risk of ATB (Table 2).

**Table 2:** Design of crude odd ratios by univariate analysis depending on cytokine response from ESAT-6 stimulated PBMC.

	Odds ratio	95% IC	p-value
Study on T-SPOT cell-free media (>7 SC/250000 PBMC)			
IFN-γ ≥100	3.0	1.4–66	0.0050
IL-4/IFN-γ x100 ≤ 30	3.9	1.6–9.7	0.0030
IL-12/IFN-γ x100 ≤ 105	7.0	2.5–19.4	0.0002
TNF-α/IFN-γ x100 ≤ 8.5	4.3	1.9–10.1	0.0007
GM-CSF/IFN-γ x100 ≤ 9.5	5.8	2.3–14.3	0.0002
Eotaxin/IFN-γ x100 ≤ 3.5	5.4	2.3–13.0	0.0001
IFN-α/IFN-γ x100 ≤ 30	4.3	1.9–9.9	0.0005

95%CI, 95% confidence interval. SC, secreted cells.

### Cytokine signatures in ATB versus LTBI groups

*Mtb*-specific T cell cytokine secretion were assessed using multiplex microbead assay dedicated to quantify 24 cytokines in cell culture supernatants. For 5 out of these 24 cytokines, concentrations quantified in less than 50% of the samples so they were excluded from analysis (IL-1β, IL-2, IL-7, IL-13 and MIG). Among the 19 remaining cytokines, 10 of them had or tend to have a significant difference between levels in supernatants from ATB versus LTBI groups. The median cytokine concentration was higher in LTBI patients for IL-5, IL-12, TNF-α, GM-CSF, Eotaxin and IFN-α whereas it was lower for IL-15, MIP-1β and IP-10 when compared to ATB patients (Table 3).

**Table 3:** Concentrations in pg/ml of cytokines secreted by PBMC after ESAT-6 stimulation in 36 patients with ATB and 115 with LTBI. LOD, limit of detection; n, number of values above detection level; nd, non determined.

	LOD	ATB		LTBI		
		n	median (IQR)	n	median (IQR)	p value
**IL-1**β	15	1	21.3	14	16 (15–19)	nd
**IL-1RA**	20	35	228 (200–365)	115	282 (207–381)	0.2579
**IL-2**	5	15	12 (7–26)	55	7 (6–11)	nd
**IL-2R**	35	35	1356 (77–208)	115	146 (104–240)	0.3860
**IL-4**	5	35	13 (10–18)	78	15 (13–18)	0.0920
**IL-5**	0.5	35	0.6 (0.6–0.9)	115	0.8 (0.7–0.9)	0.0011
**IL-6**	5	35	35 (18–80)	108	30 (13–60)	0.1797
**IL-7**	15	18	20 (16–41)	54	17 (16–38)	nd
**IL-8**	5	35	22000	115	22000	0.7270
**IL-10**	0.5	35	0.9 (0.7–1.1)	114	0.8 (0.7–1.1)	0.4981
**IL-12**	5	35	37 (30–61)	115	54 (38–75)	0.0038
**IL-13**	5	1	5	0	-	nd
**IL-15**	15	28	56 (24–151)	102	30 (22–43)	0,0077
**IL-17**	1	35	38 (35–47)	115	42 (34–59)	0.3736
**TNF-**α	1	35	3.8 (3.2–5.7)	115	4.7 (3.6–6.3)	0.0301
**GM-CSF**	0.5	35	4 (3.2–5)	115	5.8 (3.7–14.9)	0.0001
**MIP-1**α	10	35	129 (57–373)	115	132 (42–327)	0.7847
**MIP-1**β	10	35	292 (150–667)	115	184 (109–306)	0.0186
**IP-10**	1	34	77 (31–373)	115	40 (20–74)	0.0108
**MIG**	5	21	33 (15–123)	46	17 (5–61)	nd
**Eotaxin**	0.5	35	1.3 (1.0–2.1)	115	1.9 (1.4–2.4)	0.006
**Rantes**	10	35	1228 (695–1804)	115	1515 (1005–1965)	0.1033
**MCP-1**	10	35	75 (42–233)	115	86 (50–163)	0.7259
**IFN-**α	10	35	15 (11–21)	115	18 (15–26)	0.0020

*Mtb*-specific T cell profiles in ATB versus LTBI groups were then assessed using cytokine to IFN-γ ratios. Lower cytokine/IFN-γ ratios were significantly detected for 6 cytokines in ATB group when compared to LTBI group (Fig 1C, 1E and 1G and Table 2). The index medians (IQR) for ATB and LTBI were respectively: 10.5 (6.8–28.3) vs 32.05 (11.3–71.0), p<0.01 for IL-4; 47.6 (21.6–87.5) vs 115.9 (50.7–287.4), p<0.0001 for IL-12; 4.2 (2.3–8.5) vs 11.2 (IQR 4.3–22.8), p<0.001 for TNF-α; 4.6 (2.2–7.7) vs 14.4 (5.7–30.1), p<0.0001 for GM-CSF; 1.3 (0.8–3.2) vs 4.3 (1.7–9.4), p<0.0001 for Eotaxin; 12.2 (8.5–40.4) vs 46.5 (18.4–98.9), p<0.001 for IFN-α. These results were confirmed by analysis of the IL-4, IL-12, TNF-α, GM-CSF, Eotaxin and IFN-α T cell response to CFP-10 stimulation (data not shown).

**Fig 2:**
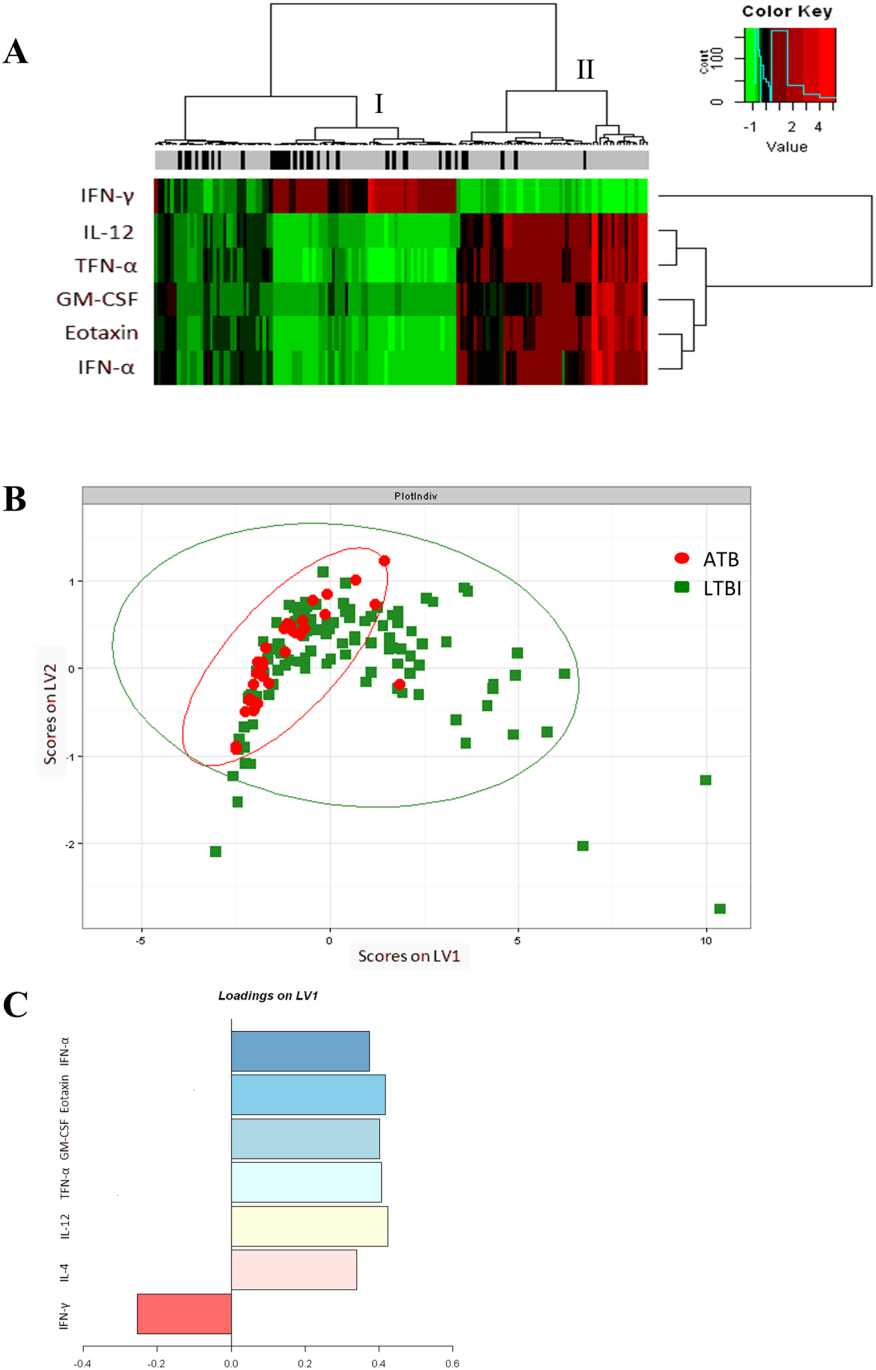
Identification of cytokine profiles associated with ATB. **A.** Unsupervised hierarchical clustering was performed according to the similarities in cytokine expression profiles which were visualised using the indicated color scale. Cytokine concentrations were indicated using a color scale that ranges from green (low) through black to red (high). The dendrogram above the heat map illustrated degrees of relatedness between the expression profiles evident within the various patients. Subjects with ATB (n = 35; black boxes on the strip above the heat-map), with high IFN-γ and low IL-12, TNF-α, GM-CSF, Eotaxin and IFN-γ secretions tended to cluster together (cluster I on the dendogram), while subjects with LTBI (n = 125; grey boxes) with IFN-γ and high IL-12, TNF-α, GM-CSF, Eotaxin and IFN-γ secretions also tended to cluster together (cluster II). The dendrogram on the right-hand side of the heat map indicates relationships between the expression profiles of the analysed cytokines across all the patients assessed in each study. **B.** Partial least squares analysis model of all 7 cytokines classified individuals with 61% overall accuracy for classification and 55% accuracy for cross-validation (red circles, ATB; green squares, LTBI). Latent Variable 1 (LV1), linear combination of the variables included in the model, LV2, orthogonal to the first combination. 95% of each population was circled. **C.** Latent variable 1 best separated individuals who are ATB from those LTBI. Cytokine loadings indicate multivariate cytokines associated with ATB.

Discrimination between ATB and LTBI was low for each of IL-4, IL-12, TNF-α, GM-CSF, Eotaxin and IFN-α when analyzing ROC curves (AUC = 0.69, 0.72, 0.70, 0.74, 0.73 and 0.70 respectively; data not shown). A threshold was determined for each cytokine by using the Youden index which defined risk groups with increase ATB odds ratio (Table 2). These risk groups were visualized by circles on biparametric graphs (Fig 1), on an unsupervised hierarchical analysis (Fig 2A) and on a PLSDA that determined multivariate combinations of all cytokines best differentiating individuals on their disease status (Fig 2B). LV1 stood for Latent Variable 1 and was a linear combination of the variables included in the model, carried out in such a way that both the covariance of X and the correlation between X and Y were maximized. After the creation of this first dimension, a second latent variable (LV2), orthogonal to the first, was constructed. LV1 of our PLSDA model best separated individuals with ATB from those who remained latently infected by TB (Fig 2B and 2C). Subjects with ATB had higher levels of cytokines positively loaded on LV1 (IL-4, IL-12, TNF-α, GM-CSF, Eotaxin and IFN-α) and comparatively lower levels of cytokines negatively loaded on LV1 (IFN-γ; Fig 2C).

## Discussion

T cell signature associated with active disease and latent forms of *Mtb* infection was characterized using a commercial IGRA, the T-SPOT assay, and cytokine analysis with a microbead multiplex assay to simultaneously measure multiple immune mediators in the cell culture supernatants. The purpose of this study was not to develop and evaluate new markers for the diagnosis of ATB or LTBI in clinical practice but to describe cytokines profiles associated with ATB and LTBI. Thus, detection of high IFN-γ release and impaired capacity of multi-cytokine secretion appeared as signatures of ATB. Both low absolute secretion (IL-12, TNF-α, GM-CSF, Eotaxin and IFN-α) and low cytokine productions relative to IFN-γ (IL-2, IL-4, IL-12, IP-10, MIG, TNF-α, GM-CSF, Eotaxin and IFN-α) could highlight a defect in TB control.

There is no diagnostic gold standard for LTBI and IGRAs are imperfect tests only intended for diagnosing *Mtb* infection based on IFN-γ detection. In our study, patients were selected based on a positive T-SPOT result since this assay appears to be more efficient than TST and probably more sensitive than QFT [18]. This selection criteria excluded patients that were tested negative regardless of their TB status (ATB or possible LTBI).

Although IGRAs are positive over an established cut-off value, we observed that IFN-γ levels above a threshold were up to seven times as frequently associated with ATB compared to values below that threshold. Previous studies in low incidence countries have demonstrated that higher quantitative IFN-γ results were associated with ATB [19,20]. In high burden settings, response was also significantly higher in ATB but it has low discriminatory ability to rule in and out disease [21–24].

We analysed additional cytokines besides IFN-γ to describe the ATB versus LTBI T cell profile in IGRA supernatants. In contrast with IGRA results, lower IL-12, TNF-α, GM-CSF, Eotaxin and IFN-α secretions were observed in ATB when compared to LTBI groups. Lower ratios corresponding to cytokine cell secretion relatively to IFN-γ production strengthened these results (IL-4, IL-12, TNF-α, GM-CSF, Eotaxin and IFN-α). This suggests impairment in multi-cytokine secretion in individuals with ATB, whereas the capacity of IFN-γ secretion could be preserved. Our observations were related to immune response against specific TB antigen, namely ESAT6 and CFP10 and may be different for other antigens.

This observed defect in cytokine response correlates with a series of recent studies highlighting that poor IL-2 functional T cell response was associated with ATB [6,25]. Lower IL-2/IFN-γ secretion [7,26] and a relative shift from IL-2 towards IFN-γ production were determined in ATB compared to LTBI or successfully treated TB subjects [5–7,25,26]. Similar observations were also made with other Th1 related cytokines, IP-10, MIG, TNF-α [5,7]. Analysis of T cell response against ESAT-6/CFP-10 at single cell level using flow cytometry were also in line with these results [13,15,27–29]. Multicytokine *Mtb*-specific T cell response is thought to protect against TB, while loss of this polyfunctionality may be the hallmark of ATB. Vaccination studies suggest that induced polyfunctional T cells have been associated with efficient control of *Mtb* infection [30–34].

Otherwise, the differences found between the cytokine concentrations and ratios, even if statistically significant, were very small and a huge overlap exists between the groups. Therefore, there is no diagnostic value. That is why these small significant differences should be confirmed in larger studies.

TB infection may be better described as a continuum composed of different equilibria between immune response and bacterial metabolism, instead of a bimodal mode including latent and active phases [35]. Thus, characterization of T cell signatures in response to *Mtb* antigen stimulation might serve in new discriminatory approaches taking into account odd ratios in a predictive model [24]. In this study, conducted in a low incidence setting, cytokine secretion to IFN-γ production ratios helped determine groups with risk of ATB ranging from 3 to almost 7 in patients tested IGRA positive. In addition, patients with LTBI categorized in such groups may also have higher risk to progress towards the disease. Thus, assessment of cytokine profile in response to ESAT-6/CFP-10 may be helpful for TB screening or to minimize long, expensive and invasive investigations.

In conclusion, analysis of multi-cytokine response in IGRA supernatants suggested that distinctive patho-physiologic profiles can be identified in ATB and LTBI. Preferential IFN-γ secretion and impairment in multicytokine *Mtb* T cells may constitute a cytokine signature of ATB.
